# The relationship between emotional intelligence and job stress in the faculty of medicine in Isfahan University of Medical Sciences

**Published:** 2014-01

**Authors:** NIKOO YAMANI, MARYAM SHAHABI, FARIBA HAGHANI

**Affiliations:** 1 Medical Education Research Center, Medical Education Department, Isfahan University of Medical Sciences, Isfahan, Iran;; 2 Isfahan University of Medical Sciences, Isfahan, Iran

**Keywords:** Emotional intelligence, Job stress, Faculty member

## Abstract

**Introduction**: health care professionals especially clinicians, undergo lots of job stress (JS). Emotional intelligence (EI) is among the variables that appear to be associated with stress. It is also included among the ways adopted by the individuals in order to resist JS in the workplace. Thus, this study aims to investigate the relationship between EI and JS in the faculty members of Isfahan University of Medical Sciences (IUMS).

**Methods**: This was a correlational study performed on 202 faculty members of IUMS. The data was gathered through two valid and reliable questionnaires (Bradberry EI questionnaire and JS questionnaire), being analyzed by SPSS software using descriptive statistics, Pearson correlation coefficient, t-test, analysis of variance (ANOVA) and linear regression analysis (α=0.05).

**Results**: 142 individuals (70.30%) filled out the questionnaires. 75% of the respondents were male and 98% were married. There was an inverse correlation between the total score of EI and the level of JS (r=-0.235, p=0.005). Moreover, among the factors of EI, self-awareness and self-management scores had significant inverse relationship with the level of JS. Linear regression analysis showed that the EI factors explained approximately 7% of the variance of JS levels of the teachers.

**Conclusions**: Individuals with high EI have less JS. Since the EI can be taught, it can be expected that the JS of faculty members can be reduced through training them on emotional intelligence. Therefore, it is recommended that short-term training courses be scheduled and designed based on the concepts of EI for teachers, particularly clinicians.

## Introduction

Working is the essence of every human being and most part of everyday lives of human being is spent on working. Working and its various aspects and effects on lives of the human beings have been investigated by many researchers. Regardless of the income, working meets a number of basic human needs such as mental and physical exercise, social bonding, self-esteem, self-confidence and feelings of competence or qualification. 

However, it may also be a major source of stress or psychological pressure (1). In psychology, stress is defined as being under psychological pressure. Stress is the physical, mental and chemical response of the human being body to the events, causing feelings of fear, excitement, anxiety, danger or anger in the individual (2).

Job stress is a pervasive problem, which affects all professional and occupational groups of the individuals in society. It causes a lot of mental and physical illness. Furthermore, it is costly to organizations and companies due to reduced performance of the employees, increased absence from work, medical costs and disability of the workers and funding for new recruitment (3).

Health workers undergo lots of job stress, especially doctors and nurses because their jobs are critical (4). For example, Ayatollahi et al. in a study titled "Evaluation of occupational hazards affecting the health of the employees in a training-health care hospital " concluded that job stress is the most common occupational problem of the physicians (with an incidence of 75%) and nurses (with a prevalence of 67%) (5). Ghulam Nejad and Nik Peyma in a study found out that the major occupational stressors for nurses include lack of receiving reward and encourage, high workload, lack of key decision makings, lack of control over working conditions, lack of career promotion (6).

Given that, faculty members of the medical schools have other key roles in addition to providing health services; therefore, even the most non-faculty physicians and nurses are exposed to occupational stressors. In the Regulations of Department of Health, titled "Overview and how to determine the duties of faculty members of the universities and medical schools and affiliated institutions of the Ministry of Health and Medical Education", the duties of the faculty members are divided into seven areas as follows: 1. Education, 2. Research and Development, 3. Personal development, 4. Administrative and management activities, 5. Providing Health care services and health promotion, 6. Specialized activities outside the university (such as participation in community and professional groups, providing scientific and technical advisory services to the community, participating in meetings) and 7. Cultural affairs. Emotional intelligence is among the variables that appear to be associated with stress. 

It is also included among the ways adopted by the individuals in order to resist the job stress in the workplace. For example, Noorian et al. in a study titled "The Effect of teaching emotional intelligence components to the doctors and nurses working in intensive care on their level of stress and anxiety” concluded that nurses and physicians experience a lot of stress and anxiety. Then, teaching the emotional intelligence components and the information relevant to emotional intelligence to the workers can be effective in coping with the stress or occupational anxiety (7).

In another study, the effect of teaching emotional intelligence skills was examined on how to deal with stress in adolescents. The results of this study showed that teaching emotional intelligence skills significantly reduced psychological stress in the adolescents (8). These studies showed that emotional intelligence should be considered in predicting job stress and other stresses as well.

Emotional intelligence is a relatively new concept employed in the management since 1990s. Emotional intelligence is a set of abilities that enable individuals to organize and manage the emotions of themselves and others. This intelligence includes understanding our own feelings and using them for taking appropriate decisions in personal and business aspects of our lives. It also determines the appropriate type of relationship that is appropriate for a profession or occupation (9). The term emotional intelligence was first introduced by psychologist John Mayer and Peter Salovi in the 1990s. They stated that the individuals who have emotional intelligence could control the emotions of themselves and others, distinguish between the positive and negative effects of the emotions and use emotional information to guide their own thought processes and personal actions. Daniel Golman, the behavioral science expert and author of "Working with Emotional Intelligence", was the first individual who introduced this concept in the organizations. Golman stated that emotional intelligence is the talent, skill, or ability, which deeply affects all individual abilities (10).

Two major models of emotional intelligence include Mayer and Salovey model and emotional-social model of Bar-On R. Mayer Salovey model has four dimensions or components including a) understanding our emotions and others’ emotions (Identifying Emotion), b) applying emotions (Using Emotion), c) the ability to perceive others' emotions (Understanding Emotion), and d) emotional Management (Managing Emotion) (11, 12). The emotional intelligence model of Bar-On R. has five components in which 15 factors are effective. The people who find higher number of these components in themselves have higher emotional intelligence than others. These factors and components include intrapersonal skills (emotional self-awareness, courage, self-esteem, self-actualization and independence), interpersonal skills (interpersonal relationships, social commitment and empathy), compatibility (problem solving, reality testing and flexibility), stress management (stress tolerance and impulse control ability) and general mood (happiness and optimism) (13).

Dehshiri in a study showed that emotional intelligence and time management significantly predict the level of job stress in the teachers. Moreover, he found out that among the emotional intelligence components, self-control, empathy and self-awareness could significantly predict the occupational stress (14). Heydari Tafresh and Delfan Azari by conducting a research showed that emotional intelligence had a significant relationship with stress coping skills (15). The study conducted by Rahim Davari showed that emotional intelligence, using problem-focused coping skills, had a significant relationship with stress (16).

Ramesar et al. in their study examined the relationship between emotional intelligence and stress management in the managers. They showed that there was a significant relationship between emotional intelligence and stress management. Moreover, according to the results of correlation and regression in the latter study, the researchers found out that stress management (the ability to resist stress or coping skills) could be considered as components of emotional intelligence (17).

Wons and Bargiel-Matusiewicz in a study titled "The relationship between emotional intelligence and stress coping in medical students" demonstrated that there was a direct relationship between increased level of emotional intelligence and the ability to cope with stressors and problems. They also noted that the individuals with high emotional intelligence had higher flexibility in dealing with the stressors (18). Finally, Arora, et al. conducted a study to explore the relationship between emotional intelligence and stress in medical students who encountered unfamiliar surgical procedures. They found out a significant relationship between emotional intelligence and stress among these students. They concluded that the students with high emotional intelligence were more willing to experience stress in unfamiliar surgical scenarios. However, compared to students with low emotional intelligence, these students had better performance as well. Finally, the researchers stated that the concept of emotional intelligence could be used to design effective stress management courses and the selection procedure of surgical residents (19).

Almost all studies showed a significant relationship between the emotional intelligence and job stress. In other words, if an individual’s emotional intelligence increases, his ability to cope with stress increases as well. In other words, those who have higher emotional intelligence experience less stress. A review of the relationship between these two variables among the faculty members of the medical school was not found. Since the relationship between these two variables can be beneficial in the educational planning and empowerment of the faculty members and even their selection procedure, the relationship between emotional intelligence and job stress in this group of individuals was studied in this study.

## Methods

This is a correlation study. It is also a cross-sectional study considering the time-interval of the study. The population consisted of all faculty members working in 2012 academic year in Isfahan University of Medical Sciences (385 individuals). Using stratified random sampling method (based on the training group), 202 individuals were selected from the faculty members. Two standard questionnaires were used to collect data. The first one was Bradbery and Grios Emotional Intelligence Questionnaire containing 28 items that measure the four components of emotional intelligence, i.e. self-awareness, self-management, social awareness and relationship management. The questions are scored in six scales including never, rarely, sometimes, often, normally, almost always or always. The score within the range of 100 to 140 shows an excellent capability; the score within 90 to 99 shows a good ability; the score within 80 to 89 may be interpreted a capability with a little reinforcement; the score within 60 to 70 should be worked on and the score within 0 to 59 is a warning to which more attention should be given. Ganji reported that this questionnaire has desirable reliability and validity (20). The second was Job Stress Questionnaire with 30 questions. This instrument was constructed by Dr. Mohammad Mossadegh Rad (2011). It measures the level of job stress of the workers in five stress dimensions including task-related stress, role-related stress, workplace-related stress, stress related to policy measures, and stress related to interpersonal relationships. The questions are scored in six scales including never, very low, low, medium, high and very high. In order to obtain a total score of job stress, the total number of responses should be divided by 30. In order to obtain the score related to one dimension of occupational stress of the workers, the total scores of stress dimensions should be divided by the number of questions of that dimension. Thus, the average score vary between one and five. Mossadegh Rad, et al. (2011) examined the validity of the questionnaire and reported the value as (82%) (21).

All the individuals who were the Faculty members of Medical Sciences in 2012-2013 academic years were eligible for this study. Those individuals with incomplete questionnaires or unrealistic demographic information were excluded from the study. All subjects volunteered to participate in this project and there was no compulsion on the participants to fill out the questionnaires. All participants were assured that their information would remain confidential. For data analysis, descriptive statistics, Pearson correlation, t-test, ANOVA and linear regression analysis were used. The software used to analyze the data was SPSS 14 (SPSS Inc, Chicago, IL, USA) (α=0.05).

## Results

Out of the 202 individuals in the sample, 142 (70.30%) filled out the questionnaires. One of them was excluded from the analysis due to unrealistic demographic data. Among the respondents, 24.8% of them were female and 75.2% of them were male. The average age of the faculty members was 46.92±7.81. The age range of the faculty members varied from 30 years old to 64 years old. Academic ranks of the respondents were distributed as follows: 4% of the respondents were instructors, 52% were assistant professors, 35% were associate professor and 9% were full professors.

The employment status of the faculty members was also as follows official - definitive (64%) and contract (20%), project (8.5%), the official test (6.4%) and tuition fee (1%). Average experience of faculty members was 15.33±8.20 years. Among these members, 17.6% were employed in eight Department of Medicine while 82.4% were employed in 21 clinical departments. 

The descriptive indices of the variables examined in this study can be found in Table 1.

**Table 1:** Mean, standard deviation, minimum and maximum scores of the subjects in the research variables

**Variable **		**Mean±SD**	**Min **	**Max**
**Job stress**		2.67±0.79	0	4
**Emotional intelligence**		90.11±12.91	60	124
**Emotional intelligence factors**	Self-awareness	20.47±3.75	7	30
	Self-management	28.32±4.80	14	41
	Social awareness	16.15±3.49	6	24
	Relationship management	25.18±4.70	9	38

As Table 1 shows among the components of emotional intelligence, self-management has the highest score (28.32) while the social awareness has the lowest score (16.15).  Pearson correlation coefficients revealed that there is an inverse significant relationship between the total score of emotional intelligence and the level of stress. In other words, if the score of emotional intelligence increases, job stress will decrease. Moreover, there was an inverse significant relationship between the component of emotional intelligence, self-awareness and self-management scores and the stress. However, no statistically significant relationship was found between the components of social awareness and relationship management and job stress (despite being negative). The results are shown in Table 2.

**Table 2:** Pearson correlation coefficients between the scores of occupational stress and emotional intelligence and its components in the faculty members

**Predictor variables **	**Occupational stress (r Pearson coefficient)**	**p**
Emotional intelligence	-0.235	0.005
Self-awareness	-0.167	0.047
Self-management	-0.269	0.001
Social awareness	-0.126	0.134
Relationship management	-0.142	0.091

As it can be seen in Table 2, the self-management component can better explain the changes in occupational stress than other emotional intelligence components.

Independent t-test showed that the mean scores of job stress and emotional intelligence have no significant differences, regarding sex and marital status (p>0.05). Moreover, the analysis of variance (ANOVA) showed that the mean score of job stress and emotional intelligence in faculty members had no significant difference with various employment status of the faculty members (p>0.05). Independent t-test showed that the mean score of job stress among clinical faculty members was significantly higher than the mean score of job stress among faculty members of applied science (p<0.001). However, the mean score of emotional intelligence and its components among clinical faculty members and applied science faculty members showed no significant difference (p>0.05). Analysis of variance (ANOVA) showed that the mean stress level among the faculty members who were full professors was significantly lower than the mean stress level among the faculty members with other academic ranks (p=0.02). Furthermore, analysis of variance showed that there was no significant relationship between scores of emotional intelligence and its components and academic ranks of the faculty members (p>0.05). 

Multiple regression analysis was used for simultaneous entry of the variables to determine the effect of each of the components of emotional intelligence in prediction of the job stress. These components include self-awareness, self-management, social awareness and relationship management. The score of job stress of the faculty members as the criterion variable of the components of emotional intelligence was entered into the regression equation as predictive variables. The results are shown in Table 3.

**Table 3:** Effect of emotional intelligence components in predicting job stress using multiple regression analysis

**Criterion variable **	**Prediction variable **	**Regression coefficients**	**t ratio**	**Level of significance**	**F**	**p**	** R^ 2 ^**
**Job stress**	**Self-awareness**	-0.16	-0.387	0.669	2.719	0.032	0.074
	**Self-management**	-0.27	-2.346	0.020			
	**Social awareness**	-0.126	-0.073	0.924			
	**Relationship management **	-0.140	-0.007	0.994			

Based on the above table, the observed F is significant (p<0.032). The emotional intelligence components approximately explain 7% of the variance of job stress factor of the faculty members. As it can be seen, the components of self-management and self-awareness are better able to predict stress-related changes. 

## Discussion

This study aimed to examine the relationship between emotional intelligence and occupational stress among faculty members of Isfahan University of Medical Sciences. The results of Pearson correlation test showed that there was a significant inverse relationship between emotional intelligence and job stress. In other words, by increasing emotional intelligence, stress should be reduced and vice versa. Therefore, the individuals who have higher emotional intelligence have the ability to withstand stressful events and situations. The results of several other studies have also confirmed these findings, including Dehshir (14), Hafezi et al. (22), Maki Poor et al. (23), Barriball et al. (24) and Birkez et al. (25). In fact, all the studies investigated found a significant relationship between emotional intelligence and job stress. This is because if an individual understands his own feelings and knows them according to the concepts of emotional intelligence, then he/she can make better choices in his/her life regarding his/her job, friends, etc. Despite the fact that the relationship is significant in the overall structure of emotional intelligence and job stress, no significant relationship was found between the components or dimensions of emotional intelligence and job stress in several studies.

Components of emotional intelligence are self-awareness, self-management, social awareness and relationship management. The components of self-awareness and self-management are related to personal capabilities of the individual while the components of social awareness and relationship management are associated with social capabilities of the individual. In this study, the relationship between self-awareness and self-management components, i.e. components relevant to personal capabilities of the individual, and job stress were significant. The results of this study are in line with the results of the study conducted by Maki Poor et al. (23). Because stress can occur when an individual is confronted with a specific event or situation that he finds challenging to his own abilities (26), thus stress is further related to the beliefs, attitudes and inner side of the individuals. Therefore, it is expected that self-awareness (the ability to understand emotions and our own strengths and weaknesses) and self-management (the ability to manage moods, stresses and our own internal capabilities) as two components of emotional intelligence (27) be better predictors for reducing stress. The result of multiple regression analysis also reflects this fact. According to this test, self-management and self-awareness are the best predictors respectively with 27% and 16% scores. These components can predict the changes related to stress level in faculty members. In Dehshir study, self-management alone was able to explain 57% of the changes in job stress factors. According to Goleman, the efficient individuals in this area (the individuals who can better manage and understand the stress) can avoid the negative emotions such as hopelessness, anxiety and irritability. These individuals face fewer difficulties in their lives or they can immediately return to favorable conditions when faced with problems and uncomfortable situations (14). These attitudes indicated that high self-control in the individuals with high emotional intelligence could protect them from the stress since it results in a situation that individuals may not encounter stressful events and if so, they can return to the ideal conditions as soon as possible.

As it was noted, the relationship between the components of social skills of the emotional intelligence, i.e. social awareness (the ability to understand individuals and groups), and relationship management (ability to create an optimal model in the others) and job stress was not significant. This finding is not in line with the results of the study conducted by Hafezi et al. (22). However, no better comparison can be made since different assessment tools of emotional intelligence such as Bar-On R., Schutte and Gelman Emotional Intelligence Questionnaires were used in various studies. Each of these tools has different dimensions of emotional intelligence. In the case of no significant relationship between the above two components and job stress, two reasons can be cited.

First, as noted above, it is more logical that there would be a relationship between the issue of work-related stress and personal empowerment component of emotional intelligence of the workers than other components. Then, lack of existence of a relationship between social capabilities of the emotional intelligence with job stress seems logical. The second reason could be due to the low power of the study; for example, the sample size is small in this research.

In this study, a significant relationship between demographic factors such as gender and marital status and emotional intelligence was not found. This finding was partly in line with the results of the study conducted by Kumar. These researchers in a study examined the relationship between demographic factors such as age, sex and educational level with emotional intelligence in polytechnic lecturers in Malaysia. They concluded that there was no significant relationship between emotional intelligence and gender and work experience; however, there was a significant relationship between emotional intelligence and age and educational level (28). In our study, a significant correlation was not found between marital status and emotional intelligence. A major reason for this difference could be due to the very low number of single individuals (n=3) in our study.

Another interesting result of this study was the fact that the mean score of job stress for the faculty members with instructor and full professor academic ranks was lower than that for associate and assistant professors. Perhaps one of the reasons can be due to greater work experience and job stability of the former group compared to the latter one.

Stressful events affect human being in terms of emotional, cognitive and physiological feelings. Therefore, the individual needs to be equipped with specific knowledge and skills that will help him deal with stress in the workplace. If the level of stress is beyond tolerance level of the individual, then it could endanger his health. Definitely controlling uncomfortable emotions is the key to emotional health and well-being of the individual in society. This study and other studies show that the people with higher emotional intelligence are more successful in coping with stress. According to Salovey et al, those with high emotional intelligence are more able to accurately understand and assess their emotional states and know how and when to express their feelings. They also can effectively regulate their mood states (29).

This study had several limitations that need to be addressed. First, the questionnaire used in this study was a self-assessment emotional intelligence questionnaire. The disadvantage of self-assessment tests is that the respondents can change their responses in order to represent a desired image of them. The second limitation of the study was the correlative study method, which imposed some limitations in the generalizability and interpretations of the results of the study.

## Conclusion

The overall results of this study showed that the individuals with higher emotional intelligence had less job stress. Among the components of emotional intelligence, two components of self-awareness and self-management were better predictors of job stress. Since emotional intelligence can be taught and acquired, it can be expected to reduce the job stress of medical school faculty by teaching emotional intelligence to them. Therefore, it is recommended that short-term training courses be designed and scheduled based on the concepts of emotional intelligence for faculty members, specifically clinical instructors. Moreover, it is recommended that, in addition to academic qualifications, the personality and emotional characteristics be considered in the selection procedure of faculty members, as well.
